# Editorial: Where to from here: Advancing patient and public involvement in health technology assessment (HTA) following the COVID-19 pandemic

**DOI:** 10.3389/fmedt.2023.1168278

**Published:** 2023-03-09

**Authors:** Janet L. Wale, Marie-Pascale Pomey

**Affiliations:** ^1^ Independent Patient Advocate, Brunswick, VIC, Australia; ^2^ Research Centre of the University of Montreal Hospital Centre, Montréal, QC, Canada; ^3^ Centre D'excellence sur le Partenariat Avec les Patients et le Public, Montréal, QC, Canada; ^4^ Department of Health Policy, Management and Evaluation, School of Public Health, University of Montréal, Montréal, QC, Canada

**Keywords:** patient and public involvement, patient partners, regulation and value assessment, COVID-19 pandemic, human rights to health care and participation, civil society

**Editorial on the Research Topic** Where to from here: Advancing patient and public involvement in health technology assessment (HTA) following the COVID-19 pandemic

## Where we have been, possibilities ahead

The COVID-19 pandemic brought with it challenges for science, vaccine development, therapeutics, and evidence-informed health care. Ethical and moral issues were at the forefront, particularly around vulnerable populations, equity, and involvement of patients and patient advocates in decision making individually and at policy level. Patient advocates and patient partners often found themselves shut out from decision-making processes in a situation where systems were overwhelmed, but at a time when advocacy and partnership were arguably most needed. Shortages of personal protective equipment (PPE) and limited knowledge of the virus meant that patients often died in hospitals without their families and significant others around them (1).

Patient advocates, patient partners and leaders however continued to be active throughout the pandemic. An example is where Australian consumer representatives, including from rural and regional areas and diverse cultural backgrounds, organised themselves to work with communities to identify inadequate or inappropriate aspects of patient care during the pandemic (2). They filled an important information and communication void and were also able to mobilize support from communities and politicians to address specific healthcare issues in local areas. In Québec, a “community of practices on patient experience and patient engagement” created a “white book” to share innovations focussing on how to maintain patient partnership at all levels of the healthcare system even in a pandemic (3).

We proposed the present Research Topic at the beginning of the COVID-19 pandemic, without foresight of the impacts it would have on communications in health care at all levels, including required regulatory and health technology and value assessment processes. And so the question “where to from here” with patient advocacy and community input continues to be relevant. Methodologies and opportunities are outlined in the included articles from around the globe.

## Maintaining the link with patients

Articles show that groups in Canada were able to recognise the strengths of their patient partners and patient experts to work together in designing communication pathways and technology-based services (Barony Sanchez et al.) as well as address policy issues and evaluation of promising interventions (Olivier et al.). Communication with diverse communities has been essential to transfer information to the public over the course of the pandemic. Barony Sanchez et al. describe how researchers in the province of Québec engaged co-creatively with patients and public partners with different backgrounds and literacy levels to develop and promote access to online health care through a “shared virtual space”. The leap to digital technology was essential because of restrictions in people's movements, particularly for those most at risk, the need for rapid access to care, and for better resource utilization. For a digital technology to be useful and effective, it has to be able to attract and actively immerse the user in its content.

## Decision-making process issues in HTA agencies

In Québec, the responsibility for managing the pandemic lay primarily with policy-making bodies, healthcare facilities, health ministries, regulatory bodies and agencies. Olivier et al. describe how the Institut national d’excellence en santé et en services sociaux (INESSS) played a key role in informing government on the evaluation of pandemic-related interventions. Ethicists, clinicians, patients and citizens with patient-partner expertise helped identify uncertainties and ethical considerations. Patient and citizen perspectives contributed to shifts in thinking for benefit-risk assessment of promising interventions, being strongly in favour of individual responsibility, shared decision between clinicians and patients, and the expression of free informed consent. Greater transparency in communications, not subject to media or political pressure, could reduce the risk of medical misinformation. Cellier expands on the principles behind patient involvement throughout health technology assessment (HTA) and value assessment of medical products within a healthcare system. These are necessary for fair and reasonable decisions. The Taiwan Division of HTA, Center for Drug Evaluation continued activities to improve patient involvement in their HTA processes, to enable effective and meaningful involvement with patients, carers and communities through online interactions, virtual meetings and cooperation with patients’ organizations (Chen et al.).

## Digital technology and accessibility

The patient and public involvement team at the UK National Institute for Health and Care Excellence (NICE) were quick to adapt to online meetings and virtual engagement (Rasburn et al.). They identified benefits, including enhanced accessibility without the need to travel, and so removal of barriers preventing participation by patients and carers. Participants could now control conference tool settings (e.g., sound, camera) and feel comfortable in their own environment. Furthermore, more people were able to attend and observe meetings. Drawbacks include restricted opportunities for networking, brainstorming, and inability to read body language and non-verbal communication.

## Communication of relevance and value of patient input

Early in the pandemic, NICE demonstrated to committee members the value of patient advocate members through a role-play exercise (Rasburn et al.). Brazilian women with breast cancer highlight the importance of understanding what clinical trial outcomes mean to patients (Silva et al.). Wale et al. describe from a patient advocate perspective how patient experiences can inform the value of medical products for HTAs. Where much effort has gone into increasing our understanding and use of patient experience data, including patient preference studies, patient reported outcome measures, and patient-focused registries. Patient experiences and knowledge are important to determine the relevance of evidence to clinical practice, democratize and build on the legitimacy of HTAs.

## Changes in research methods

Regulatory and HTA bodies concentrate on evidence from randomised controlled trials to determine overall benefits and risks for individuals. Courcelles et al. propose computerised modelling to address uncertainties in the evidence and predict effectiveness in individual patient groups in clinical care—considering epidemiology, a range of “standard of care” options, and longer-term effects.

## The COVID burden

Stresses caused by the pandemic on social and healthcare services continue to be evident, and with large numbers experiencing long-term consequences of COVID-19 infection (4). Many of the world's populations are not supported by strong health systems. In the African region, Sehmi and Wale utilised World Health Organization National Medicines Policies to highlight some of the issues in access to medical products and providing health care. In a second paper, they and other authors highlight how local manufacturing and strong regulatory lifecycle processes, with civil society involvement, could help (Wale et al.). Human rights to health care and participation in decision making at a local level are important.

The Research Topic effectively demonstrates the importance of patient advocates, patient partners and civil society during a pandemic, with mutual value to all.
